# Soluble Tumor Necrosis Factor Related Apoptosis Inducing Ligand Level as a Predictor of Severity of Sepsis and the Risk of Mortality in Septic Patients

**DOI:** 10.1371/journal.pone.0082204

**Published:** 2013-12-12

**Authors:** Ye Tian, Tianzhu Tao, Jiali Zhu, Yun Zou, Jiafeng Wang, Jinbao Li, Lulong Bo, Xiaoming Deng

**Affiliations:** 1 Department of Anesthesiology and Intensive Care, Changhai Hospital, Second Military Medical University, Shanghai, China; 2 Department of Anesthesiology, Naval General Hospital, Beijing, China; University of Cincinnati, United States of America

## Abstract

**Background:**

Tumor necrosis factor related apoptosis inducing ligand (TRAIL) as a member of the TNF gene superfamily induces apoptosis primarily in tumor cells. TRAIL also plays an important role in the modulation of inflammatory responses, especially in the process of immune paralysis. The aim of the present study was to examine soluble TRAIL (sTRAIL) levels in septic patients in an attempt to explore the association between sTRAIL level and the risk of mortality.

**Methods:**

Plasma sTRAIL levels were detected by ELISA in 50 septic patients and 20 healthy volunteers. HLA-DR expression in monocytes was detected by flow cytometry. Selective biochemical parameters were recorded, and patients were monitored in a 28-day period for mortality.

**Results:**

The mean plasma sTRAIL level in septic patients was significantly lower than that in healthy controls (16.9±8.3 vs. 68.3±8.6 pg/ml, P<0.01), and was significantly higher in 28-day survivors than those in non-survivors (19.4±9.8 vs. 13.9±4.7 pg/ml, P<0.05). Univariate analysis indicated that plasma sTRAIL level was positively correlated with monocyte and lymphocyte counts and HLA-DR expression level (r = 0.5, P<0.01; r = 0.3, P<0.05; r = 0.43, P<0.01, respectively). STRAIL level was negatively correlated with APACHE II score, BUN and age (r = −0.48, P<0.01; r = −0.29, P<0.05; r = −0.45, P<0.01, respectively). Multiple linear regression analysis indicated that the predictor of plasma soluble TRAIL level was HLA-DR expression (P<0.01).

**Conclusion:**

Low plasma sTRAIL levels were associated with immune paralysis and a high risk of mortality in patients with septic shock. sTRAIL may prove to be a potential biomarker of immune function and predict the survival of septic patients.

## Introduction

Sepsis, a systemic inflammatory response syndrome (SIRS) caused by severe infections, is one of the leading causes of admission to intensive care units (ICUs) [1]. Despite an improved understanding about the pathogenesis of sepsis in recent years, it remains a clinical challenge due to high morbidity and mortality [2]. It is estimated that over 750, 000 people suffered from sepsis and more than 210,000 of them died annually in the United States [3]. The prevailing concept of the pathogenesis of sepsis is a consequence of an overwhelming host inflammatory response to invading pathogens. Some recent studies [4] indicate that most septic patients survived during the hyper-inflammatory phase but tended to die during the stage of prolonged immunosuppression. The underlying mechanisms seem to include increased apoptosis of lymphocytes, decreased antigen-presenting capacity of monocytes and disordered apoptosis of neutrophils[5]. Given the fact that the immune function of septic patients undergoes dynamic changes during the clinical course, functional immune-monitoring assay and accurate risk assessment would be valuable to optimal care of these patients.

Tumor necrosis factor related apoptosis inducing ligand (TRAIL), a recently identified member of TNF ligand superfamily, is a type II transmembrane protein with an extracellular carboxy terminal domain [6]. Soluble TRAIL (sTRAIL) is generated by enzymatic cleavage of this extracellular domain. TRAIL induces apoptosis of susceptible cells by binding to TRAIL-R1 (death receptor 4) or TRAIL-R2 (death receptor 5), both containing the functional death domain. TRAIL can potentially interact with decoy receptors, including TRAIL-R3, TRAIL-R4 and soluble receptor OPG. Although they have no ability to transduce death signals, they may protect cells against TRAIL-induced apoptosis [6], [7].

Recent studies [8], [9] showed that TRAIL played an important role in regulating immune responses, and in vitro experiments showed that exposure to infectious HIV-1 led to the up-regulation of sTRAIL and membrane bound TRAIL in monocytes and dendritic cells. It was also found [10] that s-TRAIL increased rapidly in healthy volunteers who received a single-dose endotoxin infusion, which was normalized 6 h after drug administration. Renshaw et al [11] demonstrated that human neutrophils expressed both mRNA and protein of TRAIL, TRAIL-R2 and TRAIL-R3, and that neutrophil apoptosis was specifically accelerated by exposure to a recombinant form of TRAIL. In addition, the result of an experimental model of sepsis [12] showed that administration of recombinant TRAIL improved the innate immune response and enhanced survival in septic mice. These studies seem to support the idea that TRAIL might be involved in sepsis by regulating apoptosis of inflammatory cells and facilitating resolution of inflammation. Some other recent studies [13], [14] found that TRAIL generated by CD8+ T cell was associated with sepsis-induced immune paralysis, and that neutralization of TRAIL restored the ability to control the secondary infection in CLP-induced septic mice. Collectively, TRAIL is reported to be closely involved in the pathogenesis of sepsis but the exact regulatory pattern remains to be elucidated.

Recently, a series of studies has been published, suggesting that sTRAIL could be a biomarker for inflammation in chronic kidney disease, coronary artery disease, autoimmune disease and transplantation [15]–[19]. However, there is no study reporting the role of sTRAIL in septic patients. The aim of the present study was to determine the plasma level of sTRAIL in septic patients and explore its correlation with the risk of mortality.

## Materials and methods

### Patients and health controls

This prospective study evaluated a total of 50 septic patients who were admitted to the surgical intensive care units of Changhai Hospital (Shanghai, China) between September 2011 and May 2012. Twenty healthy volunteers (15male and 5 female) with a mean age of 50.1±7.5 years were enrolled as a control group. Written informed consent was obtained from the patients or legally authorized representatives. The study protocol was approved by the ethics committee of Changhai Hospital (CHEC2011-076).

### Inclusion criteria

Inclusion criteria were patients older than 18 years who were admitted to the ICU with a diagnosis of sepsis according to the criteria of the American College of Chest Physicians/Society of Critical Care Medicine. Sepsis, severe sepsis and septic shock were defined according to the internationally accepted criteria [20]. Patients were excluded from the study if they met one or more of the following criteria: patients who received immunosuppressive therapy, who had a history of organ transplantation, who were in a pregnant/lactating state, or with pre-existing hematological or autoimmune diseases or chronic kidney diseases, or without informed consent.

### Study design

This was an observational prospective study carried out in a single surgical ICU. The consecutive patients diagnosed with sepsis were treated according to the accepted standard treatment including antimicrobial therapy, fluid resuscitation and mechanical ventilation. Patients were screened during ICU stay and followed up for a 28-day period. Clinical and biological variables were recorded after admission. They included demographic characteristics, primary infection sources, identified microorganisms, blood routine, alanine aminotransferase (ALT), aspartate aminotransferase (AST), glucose, serum electrolytes, creatinine (Cr), and blood urea nitrogen (BUN). The severity of disease was assessed by the Acute Physiology and Chronic Health Evaluation II (APACHE II). Blood samples were collected within the first 24 h after diagnosis.

### Laboratory measurements

Blood routine, ALT, AST, glucose, serum electrolytes, Cr and BUN were measured using commercially available kits immediately after drawing the venous blood. To measure the sTRAIL, serum was separated and stored at −80°C until analysis.

TRAIL was determined with an enzyme-linked immunosorbent assay (ELISA) kit according to the manufacturer's protocol (R&D systems, Minneapolis, MN, USA). Expression of cell surface HLA-DR on monocytes was measured by flow cytometry within the first 24 h after diagnosis. Monoclonal antibodies were used as listed: CD14-APC (Clone: 61D3, eBioscience, CA, USA) and HLA-DR-PerCP-Cy5.5 (Clone: LN3, eBioscience, CA, USA). Negative controls were mouse monoclonal antibodies IgG1-APC (clone: P3.6.2.8.1, eBioscience, CA, USA) and IgG1-PerCP-Cy5.5 (clone: P3.6.2.8.1, eBioscience, CA, USA), which were isotype-matched according to the manufacturer's recommendations. Monocytes were identified and gated based on CD14 staining and side-scatter characteristics. Each sample was analyzed with more than 1,500 monocytes. The percentage of HLA-DR positive monocytes out of the total population of monocytes was recorded and analyzed.

### Statistical analysis

Quantitative data were expressed as mean ±SD or median and range, as appropriate. The Mann-Whitney test or one-way ANOVA was used in statistical analysis to compare differences between groups. Categorical data between the groups were analyzed using the chi-square test. Pearson's correlation analysis was conducted to examine the relationship between sTRAIL and other variables. Variables with a P value <0.1 in univariate analysis were included in the multivariate adjusted model. Multivariate regression analysis was then performed for the independent variables by ‘enter’ method. All the analyses were performed with SPSS software (version 17.0; SPSS Inc., Chicago, USA) and a *P* value of less than 0.05 was considered statistically significant.

## Results

### Patient characteristics

A total of 73 patients with sepsis were admitted to the ICU during the study period, of whom five patients were excluded from the study because of the age limit and 18 patients were excluded because they met at least one exclusion criterion. Finally, 50 patients (39 male and 11 female) were enrolled in this study. They ranged in age from 19 to 90 years with a mean of 66.1±15.4 years. The most common site of infection was the abdomen (82%, 41/50), followed by the lung (10%, 5/50) and urinary system (4%, 2/50). Gram-negative bacteria were identified as the main nosogenesis of infections (40%). APACHE II score within 24 h of diagnosis was 17.0±9.1. The mean mechanical ventilation time was 4.7±5.3 days, and the mean duration of ICU stay was 12 (1–33) days. The number of patients in the sepsis group, severe sepsis group and septic shock group was 16, 19 and 15, respectively. There was no age or gender difference between the three groups. The mortality in the sepsis group was significantly lower than that in the other two groups (5/16 vs. 9/19 or 9/16, P<0.05 for both, chi-square test). Twenty-three patients died during the study period, the 28-day mortality rate being 46% (Table 1).

**Table 1:** Clinical characteristics and biochemical data of septic patients.

Variables	Survivors(n = 27)	Non-survivors(n = 23)
Age	62.0±15.7	66.5±15.2
Gender(male/female)	23/4	18/5
Primary disease		
Gastrointestinal perforation	14	11
Intestinal obstruction	8	7
Pyogeniccholangitis	2	3
Primary infections site		
Abdomen	22	19
Lung	3	2
Microorganisms		
G+	3	5
G-	9	11
Fungi	3	4
Combined infection	9	6
APECHEII score	10.9±5.3	24.3±7.2**
ICU days	10(1–29)	15(1–33)*
Ventilation days	2.3±1.7	7.9±6.6**
HLA-DR	48.5±22.0	31.3±14.7**
s-TRAIL	19.4±9.8	13.9±4.7*

*P<0.05;

**P<0.01. ICU days were compared using Mann-Whitney test. APECHEII score, Ventilation days, HLA-DR and s-TRAIL were compared using one-way ANOVA.

### Plasma TRAIL levels in septic patients

**Figure 1:**
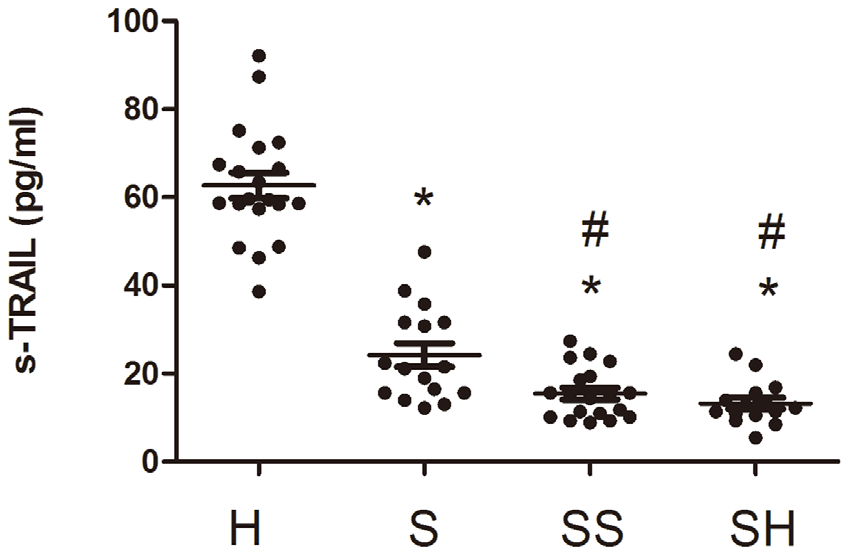
Plasma TRAIL levels in septic patients and healthy controls. *compared with healthy controls, P<0.01; # compared with the sepsis group, P<0.05. H: healthy control; S: sepsis; SS: severe sepsis; SH: septic shock.

As shown in Fig. 1, the mean plasma TRAIL level was significantly lower in the septic patients than that in the healthy controls (16.9±8.3 vs.68.3±8.6 pg/ml, P<0.01). In addition, the mean sTRAIL level in patients with severe sepsis or septic shock was significantly lower than that in patients with sepsis (both P<0.05) (Fig. 1).

### Plasma TRAIL levels and HLA-DR expression

**Figure 2:**
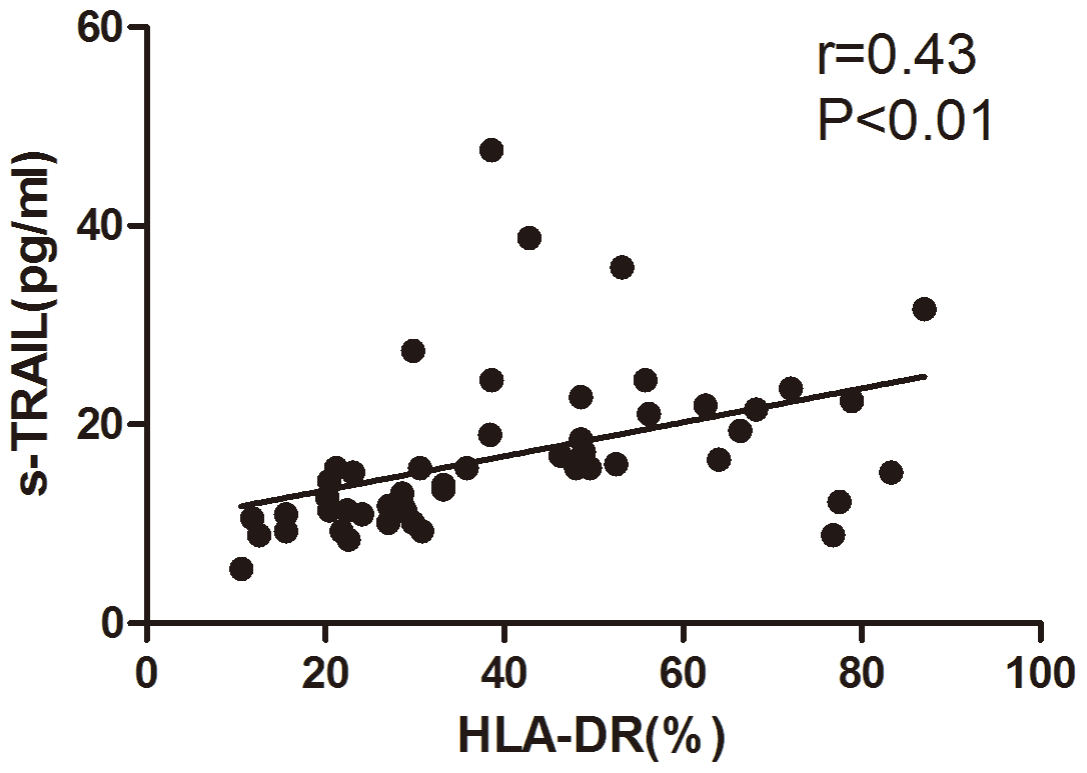
Correlations between plasma TRAIL level and HLA-DR expression on monocytes in septic patients. Pearson correlation analysis, r = 0.43, P<0.01.

To assess the immune function of the patients, HLA-DR expression on monocytes was examined. As expected, HLA-DR expression was significantly decreased in septic patients as compared with that in healthy controls (40.6%±20.7% vs. 90.7%±7.4%, P<0.01). In addition, HLA-DR expression in survivors was significantly higher than that in non-survivors during the follow-up period (48.6±22.0 vs. 31.3±14.7, P<0.01). Univariate analysis indicated that plasma TRAIL level was positively correlated with HLA-DR expression (r = 0.43, P<0.01) (Fig. 2).

### Correlations between plasma TRAIL level and other variables

Univariate analysis indicated that plasma TRAIL level was positively correlated with the monocyte and lymphocyte counts(r = 0.5, P<0.01; r = 0.3, P<0.05), while sTRAIL level was negatively correlated with APACHE II score, BUN and age (r = −0.48, P<0.01; r = −0.29, P<0.05; r = −0.45, P<0.01, respectively) (Table 2). Multiple linear regression analysis indicated that plasma TRAIL level was significantly correlated with HLA-DR expression (P<0.01) (Table 3). There was no significant correlation between sTRAIL and ALT, AST, gender, glucose, serum electrolytes, Cr, WBC count, platelet count and lipoprotein.

**Table 2:** Correlations between TRAIL and other clinical variables.

Variables	Pearson correlation	*P* value
	coefficient (r)	
Age	−0.45	0.001
BUN	−0.29	0.044
Monocytes	0.50	0.001
Lymphocytes	0.30	0.03
WBC	0.24	0.96
HGB	−0.06	0.76
PLT	0.21	0.16
ALT	0.07	0.67
AST	0.09	0.55
Cr	−0.13	0.37
Gender	−0.15	0.28

**Table 3:** Multivariate regression analysis of variables associated with s-TRAIL in septic patients.

Variables	Standardized regression	95%CI	*P* value
	coefficient		
HLA-DR	0.444	[0.068, 0.287]	0.002
Age	−0.32	[−0.33, 0.078]	0.21
BUN	−0.066	[−0.744, 0.602]	0.82
Monocytes	−0.773	[−0.39, 0.046]	0.11
Lymphocytes	−0.115	[−12.72, 9.54]	0.76

### Plasma TRAIL level predicts 28-day mortality

**Figure 3:**
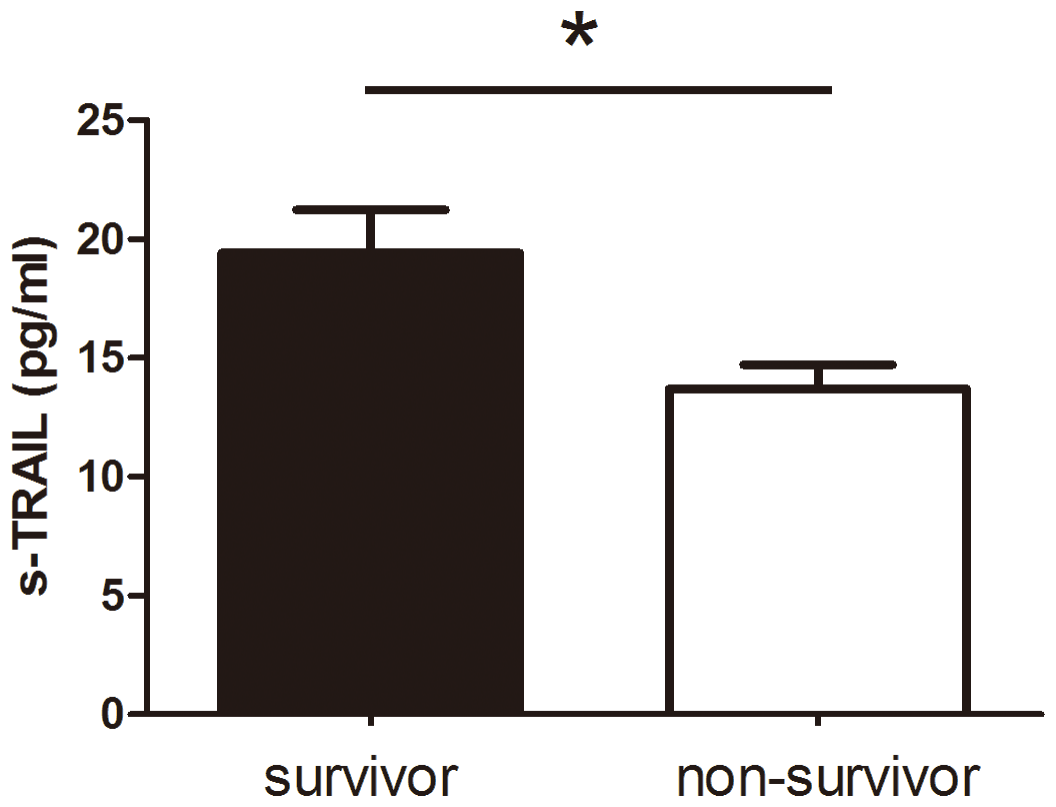
Plasma TRAIL levels in survivor and non-survivor groups. * compared with survivor group, P<0.05.

There was a significant difference in sTRAIL level when the patients were divided into a survivor group and a non-survivor group (19.4±9.8 vs. 13.9±4.7 pg/ml, P<0.05) (Fig. 3), indicating that plasma TRAIL level could be used to predict 28-day mortality in septic patients. In addition, the area under ROC curve for TRAIL used to predict 28-day mortality was 0.581(95% CI: 0.382–0.780).

## Discussion

In this study, we reported for the first time that sTRAIL was significantly down-regulated in septic patients as compared with that in healthy controls. sTRAIL level was positively correlated with monocyte and lymphocyte counts and HLA-DR expression on monocytes, and was negatively correlated with BUN and age. Low sTRAIL levels were associated with a high APECHEII score and a high risk of mortality. Polymicrobial sepsis with concomitant multiple organ failure remains one of the leading causes of death in critically ill patients. Septic insult initiates a predominant pro-inflammatory response soon after infection, and subsequently patients experience a protracted period of immune dysfunction [2]. Sepsis-induced immunosuppression is associated with impaired bacterial clearance and susceptibility to secondary infection. Septic hosts may display diverse reactions upon standard treatment, depending on their immune state. It is therefore extremely important to monitor the immune function during the course of treatment, which can not only provide some prognostic value but also guide further therapeutic decisions. TRAIL is a novel member of the TNF super family, as well as a protein functioning as a ligand that induces the process of cell apoptosis. Several studies [7] have revealed that TRAIL played an important role in mediating immune responses. Cho et al [21] found that TRAIL, together with other cytokines, facilitated DC functional maturation in response to Toll-like receptor activation. Notably, recent studies indicated that sepsis-induced immunosuppression was TRAIL dependent in a cecal ligation and puncture (CLP) model in mice. Neutralization of TRAIL was able to restore the immune activity to control secondary infections in CLP mice [14]. Thus, it would be reasonable to examine sTRAIL levels in septic patients and explore the relationship between sTRAIL level and the host immune function.

It was found in our previous study [22] that circulating monocytes from septic patients showed a markedly decreased capacity to mount pro-inflammatory response. These monocytes expressed low levels of HLA-DR, CD80 and CD86 but highly expressed programmed death ligand 1 (PD-L1). The present study found that sTRAIL level was positively correlated with HLA-DR expression on monocytes. Knowing that TRAIL plays an important role in the development of immune paralysis, our study was designed to determine TRAIL concentrations in patients with sepsis. We speculated that the low s-TRAIL level was accompanied with high OPG levels, which had been evidenced in a series of human inflammatory diseases [15], [16], [23]. As most plasma TRAIL molecules were bound by OPG, the free TRAIL was lessened. Given the fact that TRAIL expression was up-regulated in response to pro-inflammatory cytokines, another explanation might be that decreased sTRAIL level was due to the reduced production of pro-inflammatory cytokines, especially in the compromised immune status. As only one membrane receptor for TRAIL (TRAIL-R2) has been identified in mice, TRAIL regulation in humans is probably much more complex [6], [24].

It was also found in our study that the number of circulating lymphocytes was reduced significantly in the sepsis group, and that sTRAIL level was positively related with the lymphocytes count. Lymphocytes are essential regulators in acquired immunity and mice lacking T cells were succumbed more readily to septic insults [25]. Postmortem studies [5] showed that there was a dramatic loss of lymphocytes in patients who died of sepsis. Taken together, the results of the present study suggest that sTRAIL may prove to be a valuable marker of immune function in septic settings.

To confirm the prognostic value of sTRAIL in sepsis, sTRAIL levels in 28-day survivors and non-survivors were compared. It was found that survivors had significant higher levels of s-TRAIL than non-survivors. In addition, the result of univariate analysis on correlations between sTRAIL and APACHE II score, ICU stay, ventilation time, liver function and renal function showed that a strong correlation between sTRAIL and APACHE II score, suggesting that lower sTRAIL levels were related to more severe diseases, knowing that APACHE II score is one of the most popularly used severity-of-disease classification system [26]. Owing to the small sample in our study, the prognostic value of sTRAIL in sepsis remains to be clarified in larger samples of more studies.

There are several limitations in the present study. First, as we only focused on examination of sTRAIL levels within 24 h after diagnosis, we were unable to know dynamic changes in sTRAIL level. Second, the follow-up period was relatively short, and therefore we were unable to evaluate the relationship between sTRAIL and long-term outcomes. In addition, the biological effects of plasma sTRAIL were known to be largely receptor and cell type-specific, the measurement of s-TRAL would not enable us to determine the underlying mechanisms whereby sTRAIL regulates the immune response.

## Conclusion

Circulating sTRAIL may prove to be a viable biomarker of immune function in patients with sepsis. A lower level of sTRAIL was associated with immune paralysis and a high risk of mortality. Further studies are needed to better understanding the role of s-TRAIL as an apoptotic modulator and biomarker of immune function in sepsis.
